# Supplementary figures and images for: Prenatal cadmium exposure does not induce greater incidence or earlier onset of autoimmunity in the offspring
Source: PLoS One. 2021 Sep 3;16(9):e0249442. doi: 10.1371/journal.pone.0249442 (PMC8415597; doi:10.1371/journal.pone.0249442)

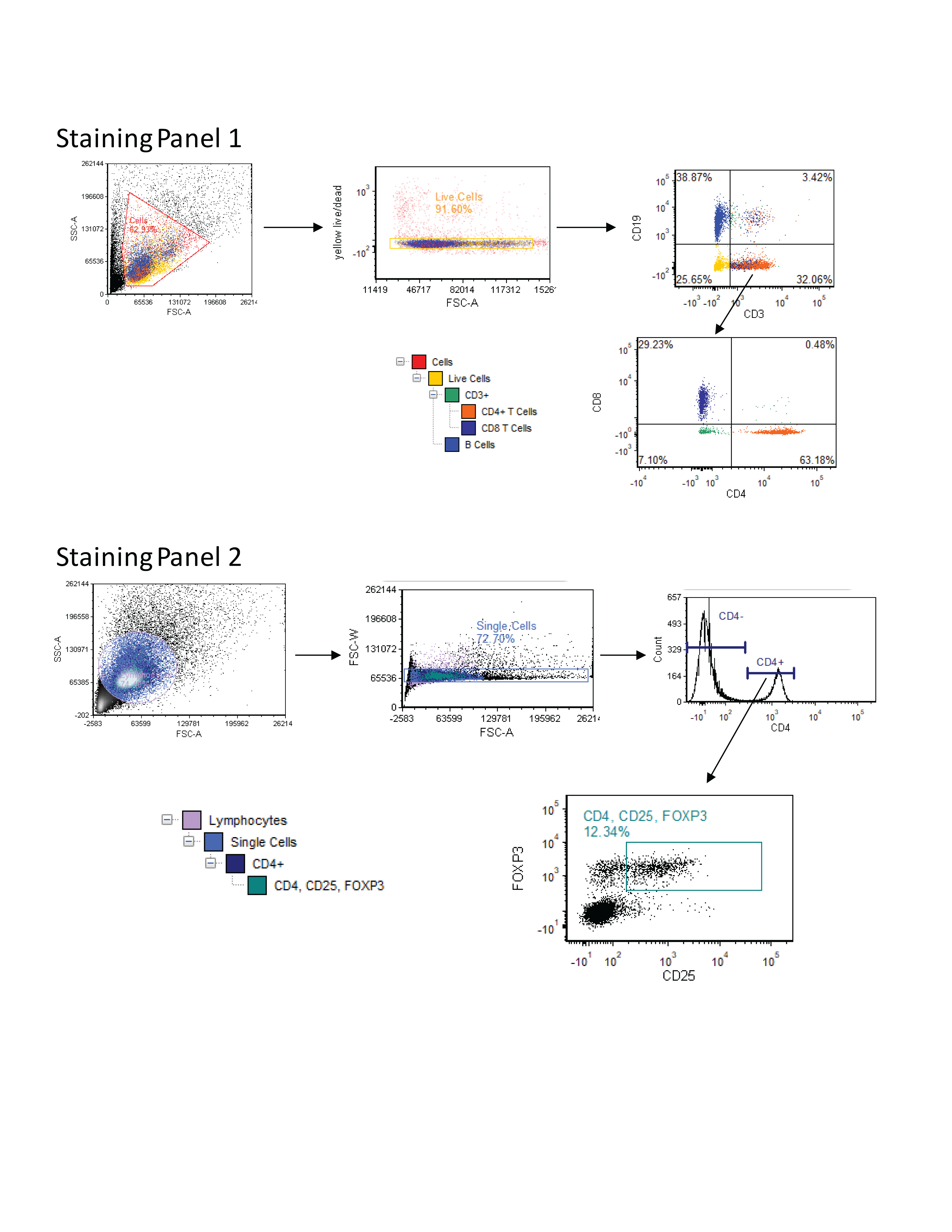

Supplement: S1 Fig — (TIF) [file pone.0249442.s001.tif]

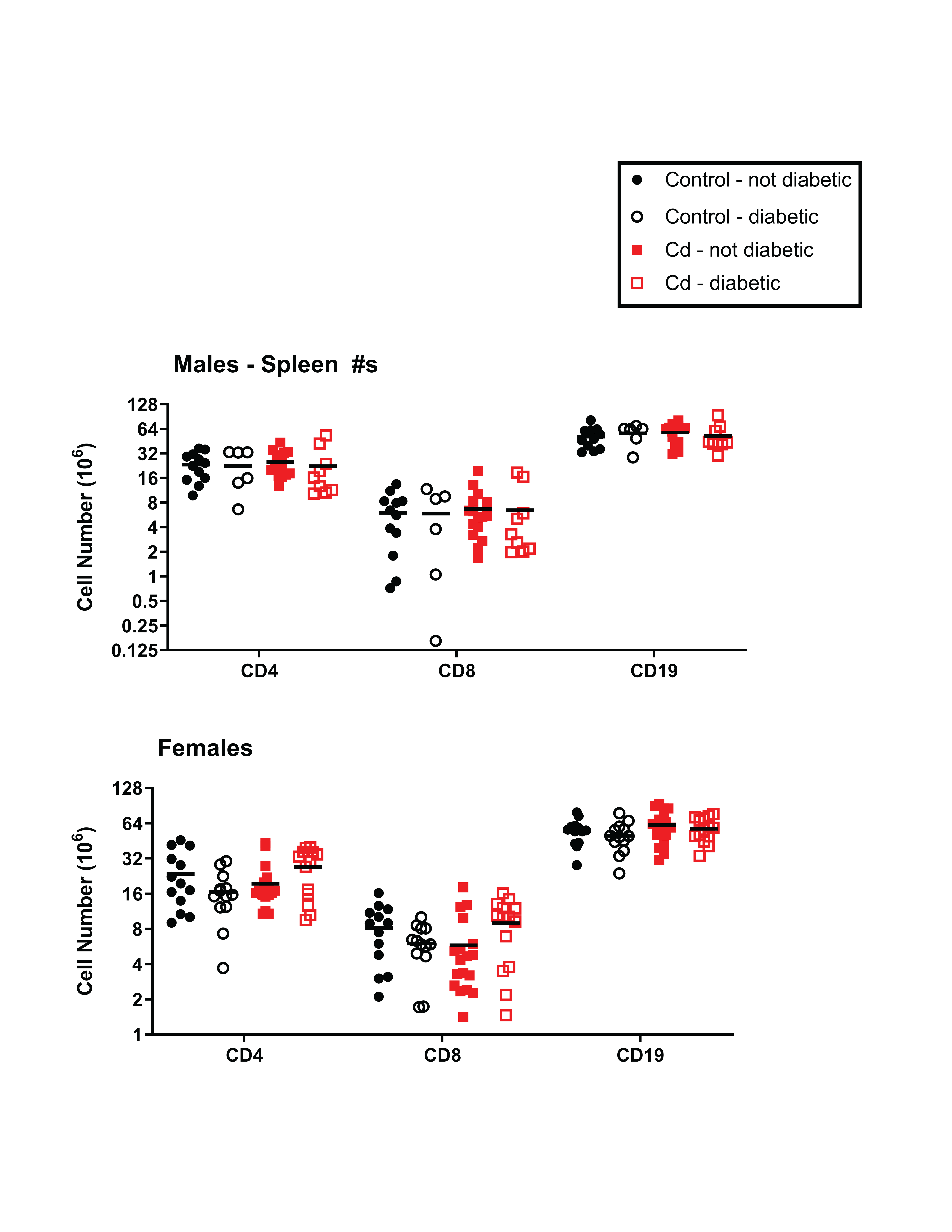

Supplement: S2 Fig — Dam and sire NOD mice were exposed to cadmium as described. At 18 weeks (females) and 22 weeks (males), the spleens were ablated and processed for flow cytometric analysis. CD4+, CD8+ and CD19+ were quantified. Symbols represent the data for individual mice and the black bar denotes the mean. Males N = 6–16; Females N = 13–19. (TIF) [file pone.0249442.s002.tif]

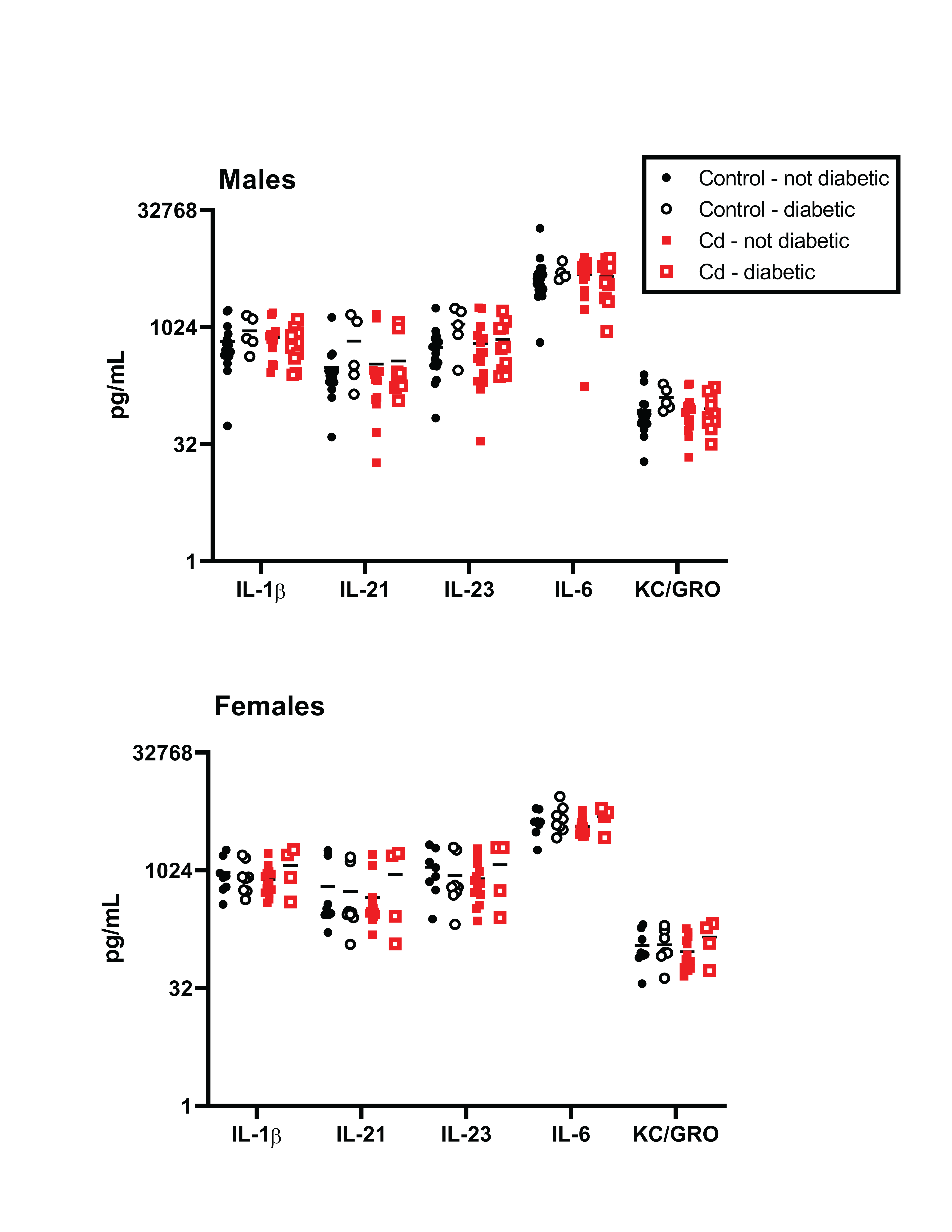

Supplement: S3 Fig — Dam and sire NOD mice were exposed to cadmium as described. At 18 weeks (females) and 22 weeks (males), the spleens of the offspring were ablated and stimulated with anti-CD3+anti-CD28 for 72h. Cytokine levels were measured using MSD multiplex plates. No differences were noted in production of these cytokines between any group. Symbols represent the data for individual mice and the black bar denotes the median. Males N = 5–17; Females N = 4–14. (TIF) [file pone.0249442.s003.tif]

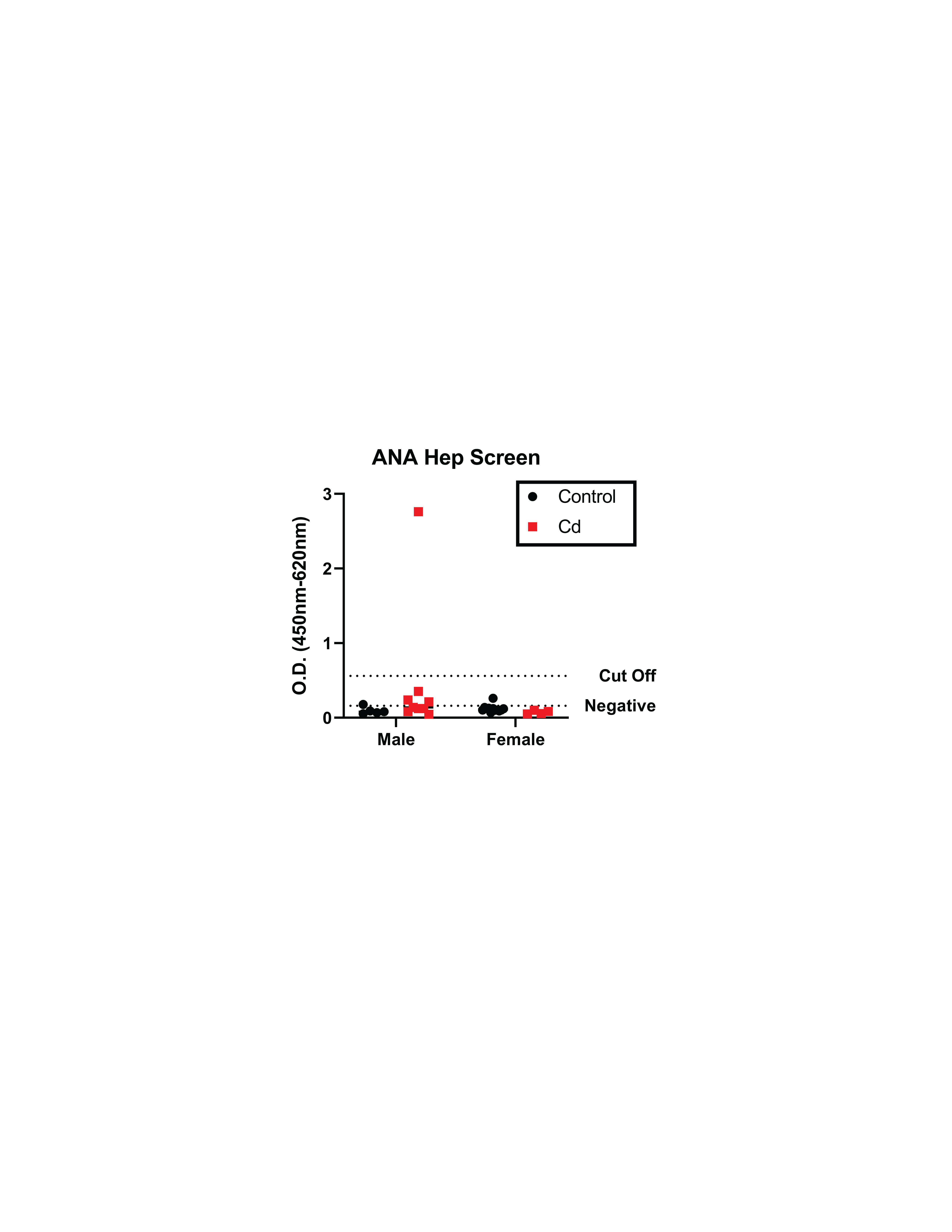

Supplement: S4 Fig — Serum autoantibodies were measured by ANA Hep Screen ELISA. (TIF) [file pone.0249442.s004.tif]

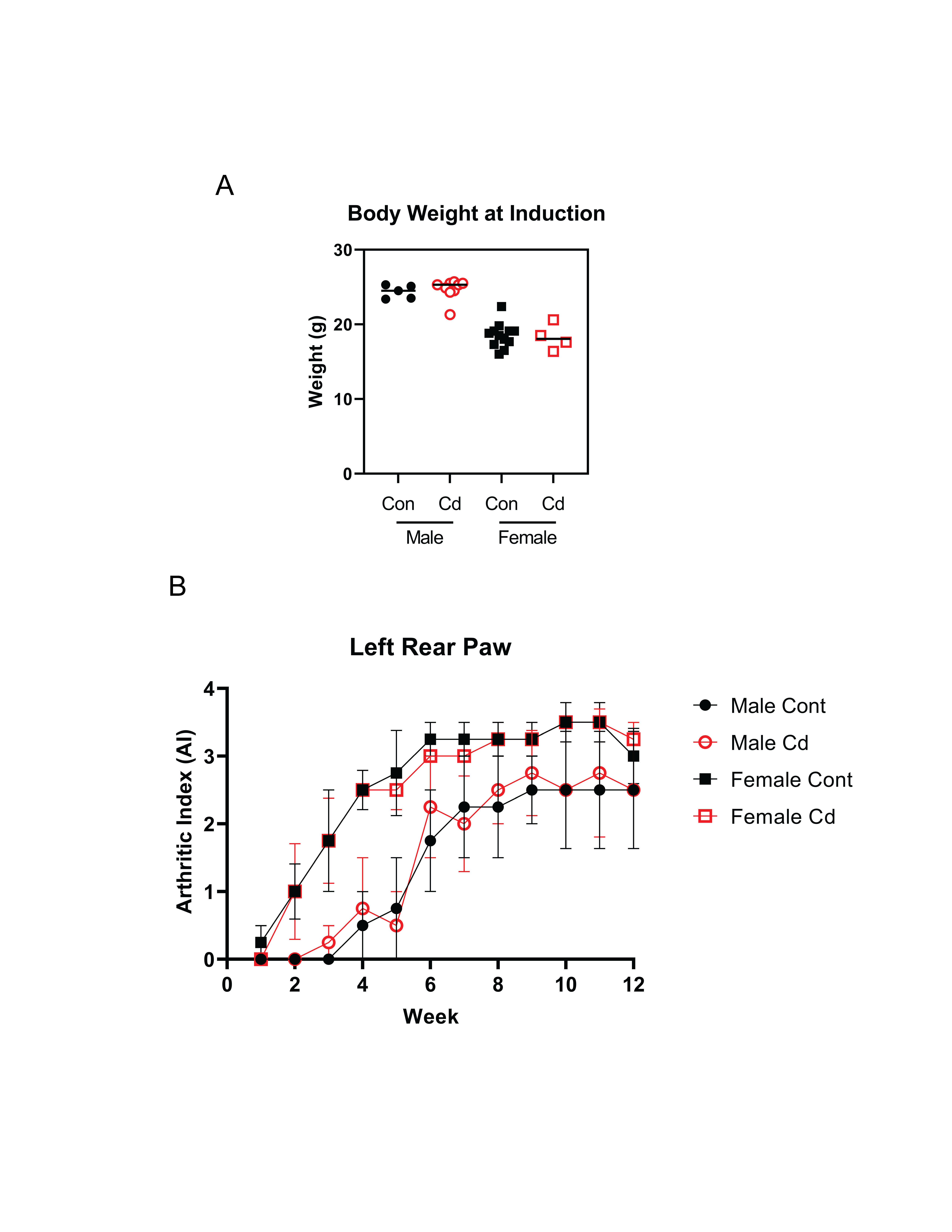

Supplement: S5 Fig — A) Body weights of male and female mice at the time of arthritis induction with zymosan A. Symbols represent the data for individual mice and the black bar denotes the median. B) Left rear paw arthritic indices. No differences were noted between control and cadmium exposure within each sex. Data are shown as mean ± SEM. (TIF) [file pone.0249442.s005.tif]

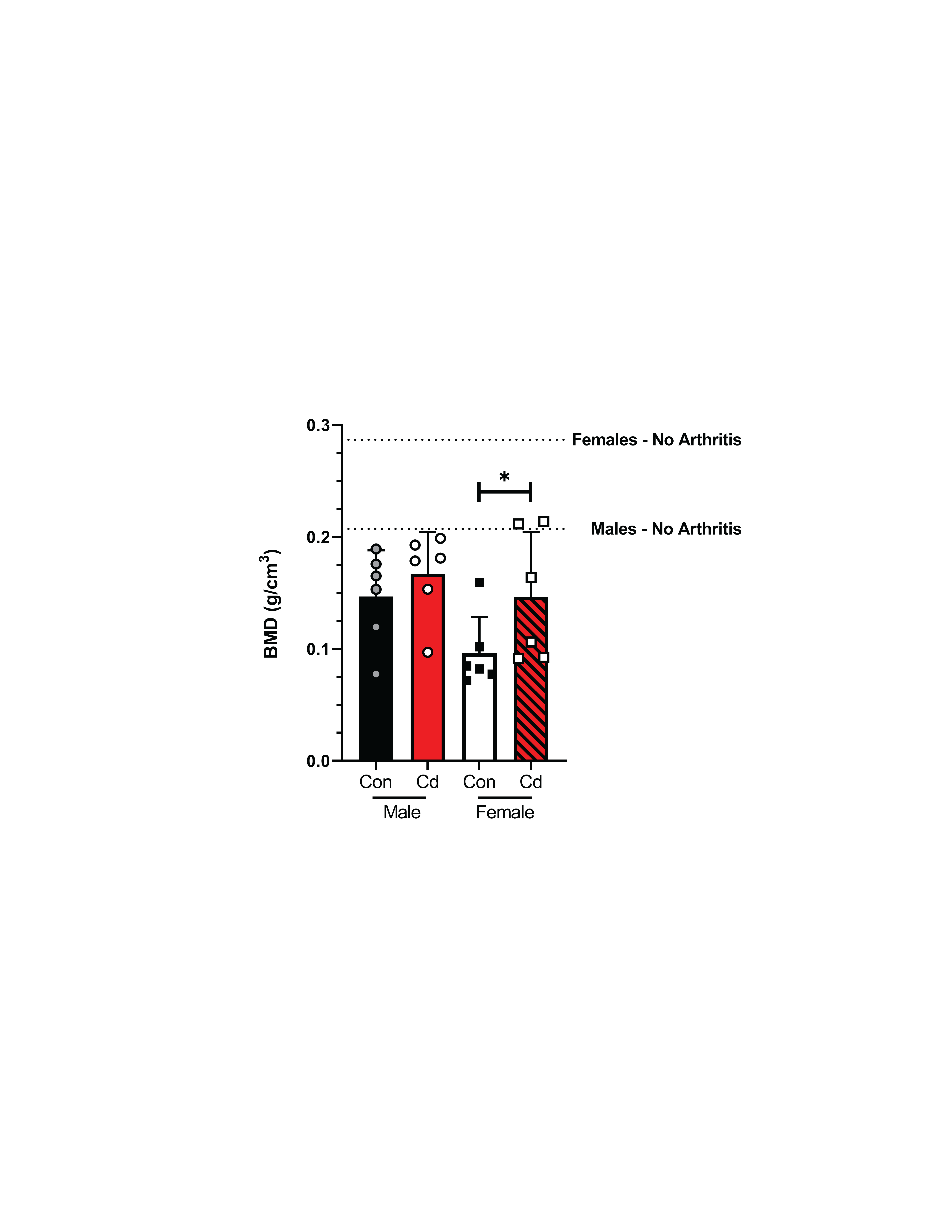

Supplement: S6 Fig — Bone mineral density (BMD) was measured at the distal femur using the same Volume of Interest (1.05 mm) following calibration with calcium hydroxyapatite phantoms of known density: 0.25 and 0.75 g/cm3. Phantoms were prepared, scanned, and reconstructed with the same parameters as Fig 7. Data are shown as mean ± SD. (TIF) [file pone.0249442.s006.tif]
